# Cognitive behavioral therapy for insomnia for individuals with multiple sclerosis (CALM): A randomized control trial protocol

**DOI:** 10.1016/j.conctc.2026.101595

**Published:** 2026-01-10

**Authors:** Catherine F. Siengsukon, Jade Robichaud, Eryen Nelson, Allison Glaser, Garrett R. Baber, Matthew K.P. Gratton, Anna Zanotto, Milind A. Phadnis, Sharon Lynch

**Affiliations:** a Department of Physical Therapy, Rehabilitation Science, and Athletic Training, University of Kansas Medical Center, Kansas City, KS, USA; b Department of Pediatrics, University of Kansas Medical Center, Kansas City, KS, USA; c Department of Biomedical and Health Informatics, University of Missouri- Kansas City School of Medicine, Kansas City, MO, USA; d Department of Psychology, University of Kansas, Lawrence, KS, USA; e Department of Occupational Therapy Education, University of Kansas Medical Center, Kansas City, KS, USA; f Department of Biostatistics, University of Kansas Medical Center, Kansas City, KS, USA; g Department of Neurology, University of Kansas Medical Center, Kansas City, KS, USA

**Keywords:** Multiple sclerosis, Cognitive behavioral therapy for insomnia, CBT-I

## Abstract

Insomnia is a common problem for individuals with multiple sclerosis (MS) occurring in at least 40 % of individuals with MS. Sleep disturbances in people with MS have been associated with a reduction in cognitive performance, physical function, psychological well-being, quality of life, and occupational function, as well as increased prevalence of fatigue, pain, depression, and anxiety. Cognitive behavioral therapy for insomnia (CBT-I), a multicomponent treatment strategy, addresses thoughts and behaviors that can negatively impact sleep and is the recommended treatment for chronic insomnia. CBT-I is shown to be more effective than pharmacological interventions long-term for treating insomnia with improvements remaining up to 10 years following CBT-I. However, there are limited studies that have examined the effect of CBT-I on sleep outcomes and comorbid symptoms in people with MS. The objective of the proposed study is to determine the efficacy of CBT-I delivered via telehealth to improve insomnia symptoms, fatigue, and health-related quality of life in people with MS. CBT-I offers a low-risk, cost-effective, non-pharmacological approach to improving sleep quality, fatigue, and daily functioning in individuals with MS. Targeting insomnia in MS may also reduce disability, enhance quality of life, increase employment rates, and lower healthcare and support costs. Furthermore, understanding factors that impact improvement in outcomes will allow more accurate individualization of insomnia treatment for people with MS.

**Trial registration:**

The CALM study is registered at: https://clinicaltrials.gov (NCT06428006).

## 1 Introduction

Insomnia is a significant concern for many individuals with multiple sclerosis (MS). A diagnosable sleep disorder occurs in approximately 50 % of individuals with MS, with up to 70 % reporting sleep disturbances [1,2]. Moreover, chronic insomnia, defined as difficulty falling asleep, maintaining sleep, or waking up too early at least 3 nights/week over the past 3 months [3], is experienced by 40 % or more of individuals with MS [4]. However, the incidence of insomnia is likely higher. One study found that only 11 % of people with MS (pwMS) who screened positive for moderate-to-severe insomnia reported being diagnosed by a health care provider [5]. Insomnia is broadly linked to reduced cognitive performance, impaired psychological well-being, and lower occupational and interpersonal functioning [6]. In pwMS, sleep disturbance compounds MS-related challenges, contributing to greater difficulties in physical functioning and activities of daily living (ADLs), heightened fatigue and pain, and more pronounced mood symptoms, ultimately lowering quality of life [[7], [8], [9], [10], [11]].

Fatigue is the most common symptom in pwMS, reported in up to 90 % of individuals [12]. In 40 % of pwMS, fatigue is reported as the worst symptom [13]. Fatigue contributes to reduced quality of life, unemployment, and decreased participation in ADL's in pwMS [14,15]. No pharmacological interventions have strong evidence for improving MS-related fatigue [16]. Not surprisingly, sleep disturbances have been associated with an increase in perceived fatigue in pwMS. A previous case series performed by Clancy and colleagues demonstrated that a cognitive behavioral therapy protocol effectively improves fatigue outcomes in pwMS [17]. These findings highlight the potential for behavioral interventions to reduce fatigue in pwMS.

Cognitive Behavioral Therapy for Insomnia (CBT-I) is a multicomponent treatment strategy that addresses behaviors and thoughts that negatively impact sleep and is the recommended treatment for chronic insomnia [18]. CBT-I improves sleep efficacy and sleep latency, reduces awakenings after sleep onset, and increases total sleep time in people with insomnia [19,20]. Furthermore, compared to pharmacological interventions, CBT-I has been shown to be more effective long-term for treating insomnia [21]. Importantly, improvements in sleep outcomes remain up to 10 years following CBT-I [22]. Prior economic evaluations consistently demonstrate that CBT-I yields favorable cost-effectiveness ratios, driven by durable symptom improvement and reductions in healthcare utilization [23]. In addition, recent meta-analyses [18,24] determined that CBT-I produced medium to large effect sizes on sleep outcomes in people with a variety of comorbid conditions. However, pwMS do not appear to have participated in any of the studies included in the meta-analyses [18,24].

While CBT-I is a first-line recommended behavioral intervention for insomnia according to guidelines from the American College of Physicians [25], the European Sleep Research Society [26], and the American Academy of Sleep Medicine [27], there are limited studies that have examined the effect of CBT-I on sleep outcomes and comorbid MS symptoms. We recently completed the first pilot randomized controlled trials to assess feasibility and preliminary efficacy of CBT-I modified specifically for pwMS [28,29]. Modifications that we made to the standard CBT-I program include: 1. For individuals who felt a nap was necessary for their health and wellbeing, the recommendation to avoid naps was modified to limit napping to ≤30 min, to take the nap as early in the day as possible, and delaying bedtime to a later time may be necessary until they were adequately tired; 2. For individuals with functional or mobility limitations or those who had concerns about falls (either individual or CBT-I provider), stimulus control was modified to perform a relaxation or distraction technique in bed; 3. For individuals who were concerned that the contraction portion of progressive muscle relaxation (PMR) might exacerbate spasticity (despite the standard instruction to gently tense muscle groups without causing discomfort), PMR was modified to focus solely on the relaxation component (i.e., releasing tension) without performing the contraction phase.

Whereas our pilot studies demonstrate feasibility and a large effect on sleep outcomes in pwMS following CBT-I [28,29], a well-designed adequately powered prospective randomized control trial (RCT) is needed to assess the efficacy of CBT-I delivered via telehealth compared to an appropriate control group to improve sleep outcomes (Aim 1) and fatigue and health-related quality of life (Aim 2). Also, determining the participant characteristics that predict improvement in sleep outcomes (Exploratory Aim 3) would help guide health care providers and individuals with MS to select an appropriate sleep intervention at an individual level.

## 2 Methods

### 2.1 Study overview

**Fig. 1:**
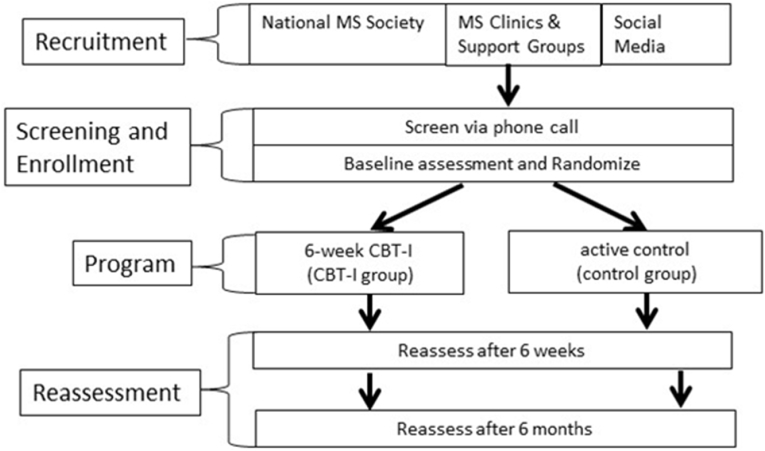
Study design.

This proposed study is a Phase II, prospective, randomized, controlled clinical trial to assess the efficacy of CBT-I in n = 70 individuals with MS (Fig. 1). Individuals meeting the inclusion/exclusion criteria are randomized to one-on-one CBT-I provided via HIPAA-compliant Zoom (CBT-I; n = 35) or active control via Zoom (Control; n = 35). The study was designed based on CONSORT criteria [30], including concealed allocation for the study coordinator who conducts screening and determines eligibility with confirmation by PI and independent, masked intention-to-treat data analysis by the biostatistician. Reassessment of outcomes is completed after the 6-week intervention and 6 months following completion of interventions.

### 2.2 Recruitment

Participants are recruited from: (1) the MS clinic at the University of Kansas Medical Center (KUMC) which serves approximately 3500 patients with MS, (2) through newsletters and emails distributed by the National Multiple Sclerosis Society to individuals across the United States, (3) newsletters distributed by the Accelerated Cure organization, (4) support groups, and (5) social media. Because this study is conducted completely remotely and with our prior recruitment collaboration with the National Multiple Sclerosis Society [28,31], we anticipate successful recruitment across the United States which will enhance generalizability of the results. Additionally, a social media campaign using targeted ad placement on Facebook is conducted in collaboration with the KUMC Office of Communications. This study is registered on clinicaltrials.gov (NCT06428006).

### 2.3 Screening procedures

Potential participants undergo a standard phone screening process conducted by the study coordinator to determine eligibility.

Phone Screen:

1. Standardized review of inclusion/exclusion criteria (Table 1).

**Table 1:** Inclusion and exclusion criteria.

Inclusion Criteria	• 18–65 years old (upper age limit in place to reduce heterogeneity in sleep architecture changes due to aging) • Diagnosis of relapsing-remitting or secondary progressive MS based on established guidelines [56] and verified by their neurologist • Mild-to-moderately severe disability (≤6 on Patient Determined Disability Steps (PDDS) scale) [32] • Report of difficulty falling asleep, maintaining sleep, or waking up too early at least 3 nights/week for the past 3 months with significant distress and impact on function despite adequate opportunity for sleep and not due to other sleep disorders as indicated in the DSM-5 • ≥10 on Insomnia Severity Index [35] • English speaking • ≥ 31 on modified Telephone Interview of Cognitive Status [36] • Has a high school diploma or equivalent to serve as a proxy measurement of reading ability to ensure adequate reading ability to participate in the study • Report having access to internet service or a data plan and access to a computer, tablet, or smart phone
Exclusion Criteria	• Known untreated sleep disorder (such as sleep apnea or restless legs syndrome) • >3 on STOP BANG indicating increased risk of sleep apnea [37] • Restless legs syndrome as determined by RLS-Diagnosis Index [39] • Circadian rhythm sleep-wake disorder as determined by the Sleep Disorders-Revised [40] • Parasomnia as determined by the Sleep Disorders-Revised [40] • Currently taking benzodiazepines, non-benzodiazepines, or melatonin supplements or agonists for insomnia • Score of ≥20 on the Patient Health Questionnaire (PHQ-9) indicating severe depression or endorsement of suicidal ideation (answer 1, 2 or 3 on #9 of the PHQ-9) [41] • Score of ≥15 on the Generalized Anxiety Disorder (GAD-7) indicating severe anxiety [42] • Current or history (up to 2 years) of alcohol or drug or alcohol abuse as indicated by DSM-5 criteria • History of other nervous system disorder such as stroke or Parkinson's disease • Currently pregnant or intending to become pregnant in the next 6 months. • Severe mental illness such as schizophrenia or bipolar disorder • Severe neurological or sensory impairments that would interfere significantly with testing • Relapse and/or corticosteroid use in the past 8 weeks • History of (within 5 years) or currently conducting overnight shift work including hours of midnight-4am • Currently receiving a behavioral sleep health intervention
2. Disability will be assessed by the Patient-Determined Disability Steps (PDDS) [32] scale which is a single-item 9-point scale ranging from “normal” (score of 0) to “bedridden” (score of 8). The PDDS has been validated and highly associated with the Expanded Disability Status Scale [33]. A score of ≤6 is considered mild-to-moderately severe disability.
3. The Insomnia Severity Index (ISI) [34] which is a valid and reliable measure of insomnia severity and consists of 7 questions each rated on a 0–4 scale. The range of scores on the ISI is 0–28, with ≥10 suggesting clinical insomnia [35].
4. The 13-item modified Telephone Interview of Cognitive Status (TICS-M) [36] will be used to determine global cognitive status. A score 31 or greater will be considered non-demented [36].
5. The 8 item STOP BANG [37] will be used to assess for risk of obstructive sleep apnea. A score of >3 is highly sensitive in detecting obstructive sleep apnea in various patient groups [38].
6. The RLS-Diagnosis Index [39] will be used to screen for restless legs syndrome.
7. The Structured Clinical interview for Sleep Disorders-Revised (SCISD-R) [40] will be used to screen for presence of circadian rhythm sleep-wake disorders and parasomnias.
8. Depressive symptoms will be assessed using the 9-item Patient Health Questionnaire (PHQ-9), with a score of ≥20 suggesting severe depression [41].
9. Anxiety symptoms will be assessed using the 7-item Generalized Anxiety Disorder (GAD-7), with a score of ≥15 indicating severe anxiety [42].

### 2.4 Baseline evaluation

Eligible individuals are emailed a link to complete the consent document and baseline assessment questionnaires using the Research Electronic Data Capture (REDCap) web-based, electronic data capture tools hosted on a secure, HIPAA compliant server at KUMC [43]. Participants are mailed the actigraph (wGT3X-BT®, LEAP ®, ActiGraph corp. Pensacola, FL) and instructed to wear the actigraph on their non-dominant wrist for 7 days and nights. Baseline assessment is completed within 2 weeks of screening. It is important to note that actigraphy is known to underestimate sleep onset latency (delayed sleep onset is one characteristic of insomnia), and there are inconsistent results in studies that attempted to use actigraphy to distinguish between those with and without insomnia. Therefore, actigraphy is not a valid assessment of insomnia and is not used as an inclusion/exclusion criterion. However, actigraphy is sufficiently sensitive to detect changes in sleep patterns modified by CBT-I and is a well-used assessment of intervention efficacy. Using actigraphy in combination with self-report assessments provides a more robust assessment of change in sleep due to CBT-I.

*Allocation concealment:* To reduce the risk of bias, the research assistant who completes actigraph analyses is unaware of the condition to which participants were randomized. While participants are aware of the group they are randomized into, they are informed that the purpose of the study is to determine the effect of two different interventions on sleep to reduce the impact of expectation bias.

*Randomization:* Participants were randomized using a permuted block randomization scheme with a fixed block size of 10 to ensure balance between groups over time. A random/pseudo-random number generator was used in SAS v9.4 to develop the randomization list. The study biostatistician wrote the program code in SAS to derive and maintain this list. The randomization list is securely stored on the study coordinator's password-protected computer, inaccessible to the research assistant conducting assessments. Following completion of baseline assessments, the study coordinator conducts informs the participants of their assigned study arm via a scheduled phone call and documents group assignment in REDCap for study tracking.

### 2.5 Primary and secondary outcomes (Table 2)

*Sleep Outcomes:* To determine if CBT-I in individuals with MS improves insomnia symptoms and sleep quality (Aim 1), insomnia severity is assessed using the Insomnia Severity Index (ISI) [35], sleep quality is assessed using the Pittsburgh Sleep Quality Index (PSQI) [44], and actigraphy is used for objective assessment of sleep outcomes:

1.The Insomnia Severity Index (ISI) [35] is used to assess insomnia symptoms. The ISI is used to determine eligibility (described above under “Screening Procedures”). Because the ISI determines insomnia symptoms for 2 weeks and baseline assessment is completed within 2 weeks of screening procedures, the ISI from screening is used as the baseline assessment of insomnia symptoms.

2. Self-reported sleep quality is indexed using the Pittsburgh Sleep Quality Index (PSQI) [44]. The PSQI consists of 9 items within 7 sleep categories and is a well-validated and reliable measure of sleep quality. The 7 sleep category scores are summed to form a single global score ranging from 0 to 21, with a global score of >5 reflecting poor sleep quality [44].
3. Participants wear an actigraph on their non-dominant wrist for 7 days and nights. An instructional handout is mailed with the actigraph detailing proper fit and safety information (i.e. remove the actigraph if there is skin irritation or if increases difficulty sleeping). A postage-paid envelope is included for participants to return the actigraph. The outcomes of interest for actigraphy are sleep efficiency and total sleep time, and we will explore wake after sleep onset, number of awakenings, sleep latency, time in bed, and sleep duration variability.

*Fatigue and Health-related Quality of Life Outcomes:* To determine if CBT-I in individuals with MS improves fatigue and health-related quality of life (Aim 2), fatigue is assessed using the Modified Fatigue Impact Scale (MFIS) [45] and the Fatigue Severity Scale (FSS) [46] and health-related quality of life is assessed using the Multiple Sclerosis Impact Scale (MSIS-29) [47].

1. The MFIS assesses the impact of fatigue on daily activities for the previous month. The MFIS consists of 21 items with 3 subscales: physical, cognitive, and psychosocial. Scores ranging from 0 to 84, a higher score indicates a greater impact of fatigue.
2. The FSS consists of 9 questions and assesses the impact of fatigue on activities for the week prior. The mean of the 9 scores is calculated, ranging from 0 to 7. The MFIS and FSS are both valid and reliable measures of self-report fatigue [48].
3. The MSIS-29 assesses health-related quality of life in the past 4 weeks, consisting of 29 with subscales of physical (20 items) and psychological (9 items). The total score ranges from 0 to 100, and higher scores indicate worse quality of life due to physical and physiological impacts of MS.

*Secondary Outcomes:* To explore the characteristics of participants that predict improvement in sleep outcomes (Exploratory Aim 3), disability (assessed by the Patient-Determined Disability Steps; PDDS) [32], depression (assessed by the Patient Health Questionnaire; PHQ-9) [41], anxiety (assessed by the Generalized Anxiety Disorder Assessment; GAD-7) [49], sleep self-efficacy (assessed by the Sleep Self-Efficacy Scale; SESS) [50], and adherence to the intervention are also assessed. Disability level, depression, anxiety, and sleep self-efficacy have a known bidirectional relationship with sleep and fatigue and are plausible predictors of responders and non-responders to CBT-I.

1. Patient-Determined Disability Steps (PDDS) [32] scale is used to assess disability. It consists of a 9-point scale ranging from “normal” (score of 0) to “bedridden” (score of 8). The PDDS has been validated and highly associated with the Expanded Disability Status Scale [33].
2. Patient Health Questionnaire (PHQ-9) [41] is a valid and reliable method to assess depression during the past 2 weeks. It consists of 9 items with a score ranging from 0 to 27. A tenth question that is not included in the summary score assesses how depressive symptoms affect functional level. The PHQ-9 is used as a screening tool and an outcome measure.
3. Generalized Anxiety Disorder Assessment (GAD-7) [49] is a valid and reliable method to assess anxiety over the past 2 weeks. This questionnaire consists of 7 items, and the score from each item is summed for an overall score ranging from 0 to 21 with a higher score indicating a higher level of anxiety. An additional eighth question assesses if anxiety impacts daily activities and sociability.
4. Sleep Self-Efficacy Scale (SESS) [50] is a 9-item self-report questionnaire used to measure sleep self-efficacy. The respondents indicate confidence in ability to accomplish 9 sleep behaviors on a 5-point Likert scale with 1 = “not confident” at all to 5 = “very confident”. The score ranges from 0 to 45 with a higher score indicating greater sleep self-efficacy.
5. Adherence to CBT-I intervention: To assess adherence to the CBT-I intervention, the sleep log is used to assess number of mornings per week the participant got out of bed at agreed upon time, number of times got out of bed if unable to sleep within 20 min, and number of nights per week the participant conducted wind-down routine. A total percentage will be calculated and used as the outcome of interest.

### 2.6 Reassessments

**Fig. 2:**
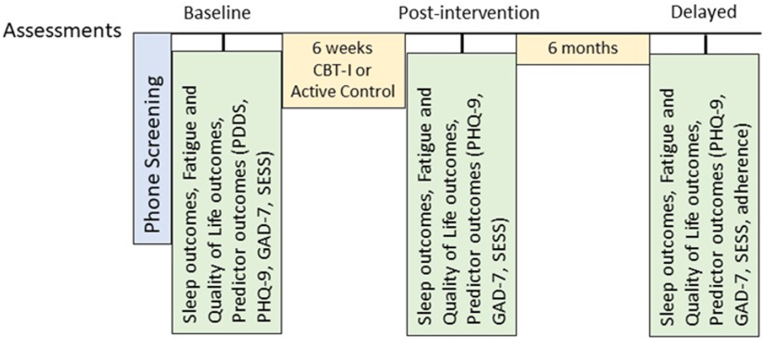
Overview of study protocol by assessments time points.

Participants undergo reassessment of the primary and secondary outcomes (Table 2) after the 6-week intervention and 6 months following completion of interventions (Fig. 2). Similar to the baseline assessment, participants are emailed a link to complete the assessment questionnaires and mailed the actigraph with a postage-paid envelope to return the actigraph.

**Table 2:** Primary and secondary outcomes.

Sleep Outcomes: (Aim 1)
1. Insomnia Severity Index (ISI) [35] (primary outcome)
2. Pittsburgh Sleep Quality Index (PSQI) [44]
3. Actigraphy
Fatigue and Quality of Life Outcomes: (Aim 2)
1. Modified Fatigue Impact Scale (MFIS) [45]
2. Fatigue Severity Scale (FSS) [46]
3. Multiple Sclerosis Impact Scale (MSIS-29) [47]
Secondary Outcomes: (Exploratory Aim 3)
1. Patient-Determined Disability Steps (PDDS) [32]
2. Patient Health Questionnaire (PHQ-9) [41] to assess depression
3. Generalized Anxiety Disorder Assessment (GAD-7) [49]
4. Sleep Self-Efficacy Scale (SESS) [50]
5. Adherence to CBT-I protocol (*Gathered from sleep log*: number of days/week got out of bed at agreed upon time, number of times got out of bed if unable to sleep within 20 min, number of nights/week conducted wind-down routine; a percentage will be calculated)

### 2.7 Study arms

#### 2.7.1 Cognitive behavioral therapy for insomnia (CBT-I)

The CBT-I program is a 1x/week, 6-week one-on-one program via video conferencing (HIPAA-compliant Zoom). The program is based on standard CBT-I manuals [51,52]. Participants maintain a sleep diary during the program to aid in tailoring the program. Each session includes a summary and graphing of sleep diary data, an assessment of treatment gains and adherence, and determination if upward titration of total sleep time is warranted. The general session outlines are as follows with each session lasting 45–60 min:

*Session 1*: subjective sleep history and description of current sleep issues, education regarding physiological mechanisms of sleep using the two process of model of sleep [53] and 3P model of insomnia [54], and tailored sleep promotion techniques, collaboratively set time in bed restriction, discuss strategies for stimulus control (if fall risk, perform counter-control in bed instead), discuss avoiding/limiting naps (limit napping to ≤ 30 min, to take the nap as early in the day as possible, and delaying bedtime to a later time may be necessary until they were adequately tired).

*Session 2*: review sleep promotion techniques, introduce diaphragmatic breathing and deep breathing as relaxation techniques to reduce pre-sleep arousal.

*Session 3*: introduce mindfulness as a technique to reduce pre-sleep arousal.

*Session 4*: introduce progressive muscle relaxation (PMR) as relaxation technique to reduce pre-sleep arousal (if concern about contraction portion of PMR may increase symptoms or spasticity, recommend only performing relaxation strategies).

*Session 5*: cognitive restructuring for negative sleep beliefs as needed, reinforce and/or continue to tailor various techniques.

*Session 6*: discuss relapse prevention and maintenance of sleep gains.

#### 2.7.2 Active control group (control)

The control group matches the intervention group for time, contact with research personnel, and delivery method. The control group receives basic sleep hygiene and lifestyle education which is very commonly used as the control for CBT-I. Importantly, participants in the control condition do not receive active components of CBT-I (i.e. time in bed restriction, stimulus control, cognitive restructuring, relaxation strategies, psychoeducation) or strategies to change sleep behavior (i.e. motivational interviewing, goal setting for sleep). The control program is a 1x/week, 6-week one-on-one program via video conferencing (HIPAA-compliant Zoom). The general session outlines are as follows with each session lasting 45–60 min and includes starting the session with basic stretches and ending the session with same stretches:

Session 1: education on sleep stages.

Session 2: education on sleep hygiene (focus on environment of temperature, noise, light, optimal sleeping positions).

Session 3: education on sleep hygiene (focus on stimulants/depressants of caffeine, medications, nicotine, alcohol, screen time).

Session 4: education on sleep hygiene (focus on diet/nutrition of large/spicy meals, fluid intake, basic diet recommendations).

Session 5: education on sleep hygiene (focus on exercise).

Session 6: discuss remaining questions.

## 3 Statistical plan and data analysis

### 3.1 Sample size justification

We will enroll 70 participants (35 in each of the two groups) in our study. Our sample size calculations are guided by effect size estimates for the primary outcome of ISI and PSQI from our pilot studies [28,29]. We hypothesize that CBT-I will perform better than the Control group for ISI and PSQI. Our hypotheses are – $H_{0}:\mu_{CBT-I}\leq \mu_{Control}$ vs $H_{0}:\mu_{CBT-I}>\mu_{Control}$ where the μ represents the mean scores of the two arms at 6 week and 6 month assessment after adjusting for the baseline.

Our calculations are purposely done in a conservative way considering the following two limitations of our pilot study comparing CBT-I to an Active Control and to a brief education group (BE) – {i} Our preliminary study was a pilot study with only 10 subjects in the CBT-I, Active Control and BE arms, and {ii} There was no significant difference in the Active Control vs BE arm for both the primary and secondary outcomes in our pilot study. Thus, we have made the following assumptions in the power analysis calculations for the proposed study – {i} The higher of the mean change scores of the Active Control and BE arm in the pilot study is considered as $\mu_{Control}$, {ii} The mean change score for CBT-I in the pilot study is considered as $\mu_{CBT-I}$, {iii} The higher of the two standard deviations (of effect size) for the CBT-I and Control group is used as the common standard deviation, {iv} sample size calculations are done with a two-sided test, with type I error set at 5 % and target power is taken as 90 %.

A ‘*Repeated Measures design’* with autocorrelation of ρ=0.5 (between the 6 week and 6 month assessment) and a type I error of 5 % was used to conduct the sample size calculations. With the assumptions mentioned above, for the *primary outcome of ISI*, a sample size of 17 in each of the two arms (n = 34 total) is required to detect a $\mu_{CBT-I}$ value of 13.8 versus $\mu_{Control}$ value of 8.1 (with common standard deviation σ=5.8) with 90 % power. Analogously, in the case of *primary outcome of PSQI*, a sample size of 25 in each of the two arms (n = 50 is required to detect a $\mu_{CBT-I}$ value of 6.7 versus $\mu_{Control}$ value of 4.6 (with common standard deviation σ=2.6) with 90 % power. Thus, max (34, 50) = 50 is chosen. The attrition rate from our prior preliminary efficacy trials of CBT-I in people with MS were: 16.7 % in-person CBT-I [28], 9.1 % in brief education [28], and 0 % in tele-health delivered CBT-I [29]. To be cautious, we will anticipate an attrition rate of 25 % due to the delayed reassessment, and so 50/0.75 ≈ 67 (rounded up to 68) is chosen. Looking at overall capability at enrolling participants, we have decided to enroll a total of 70 subjects in our study. We used the *‘Test for Two Means in a Repeated Measures Design’* platform in PASS 2020 to conduct our sample size calculations.

### 3.2 Statistical Plan

We will conduct an intention-to-treat analysis for each outcome as well as for those who complete the delayed reassessment. Descriptive statistics will be reported using mean and standard deviation for continuous variables and with frequency counts for categorical variables. The study biostatistician will not be aware of group allocation and will use the SAS statistical software to run all analyses.

#### 3.2.1 Aim 1: To determine if CBT-I in individuals with MS will improve insomnia symptoms and sleep quality

*Hypotheses*: CBT-I will improve insomnia symptoms (primary outcome; measured using the Insomnia Severity Index) and sleep quality (measured using the Pittsburgh Sleep Quality Index) and will increase sleep efficiency and total sleep time (measured by actigraphy) compared to active control after intervention and after 6 months.

*Statistical Plan*: A repeated measures model (RMM) with two groups will be used to analyze the primary outcome of ISI and secondary outcome of PSQI at 6 weeks and 6 months. The analysis will be done adjusting for baseline as a covariate. With only two time points, the analysis is simplified (compound symmetry structure) and will be conducted using a type I error of 5 %. If there is a significant between group difference, we will use the pairwise comparison from the RMM to compare the two groups at 6-weeks and at 6 months. Point estimates of the mean scores for the two groups at all time points will be reported along with the corresponding 95 % confidence interval and p-values.

#### 3.2.2 Aim 2: To determine if CBT-I in individuals with MS will improve the MS symptom of fatigue

*Hypotheses*: CBT-I will improve fatigue (measured using Fatigue Severity Scale and Modified Fatigue Impact Scale) and health-related quality of life (measured by the Multiple Sclerosis Impact Scale) compared to active control after intervention and after 6 months.

*Statistical Plan*: The same statistical analysis plan used for Aim 1 will be used in Aim 2. A RMM with two groups will be used to analyze the secondary outcomes of MFIS, FSS and MSIS. The analysis will be done adjusting for baseline as a covariate. With only two time points, the analysis is simplified (compound symmetry structure) and will be conducted using a type I error of 5 %. If there is a significant between group difference, we will use the pairwise comparison from the RMM to compare the two groups at 6-weeks and at 6 months. Point estimates of the mean scores for the two groups at all time points will be reported along with the corresponding 95 % confidence interval and p-values.

#### 3.2.3 Exploratory Aim 3: To explore the baseline characteristics of participants that predict improvement in sleep outcomes

Hypothesis Baseline disability level, anxiety, depression, and sleep self-efficacy will predict responders and non-responders (i.e. higher disability, anxiety, and depression, lower sleep self-efficacy, and lower intervention adherence will predict non-responders; lower disability, anxiety, and depression, and higher sleep self-efficacy and level of intervention adherence will predict responders).

*Statistical Plan*: For Aim 3, the outcome is dichotomous (responders vs non-responders) and a logistic regression analysis will be conducted modeling responder status (defined as meeting the minimal clinically important difference of ≥ 6 points on the ISI) at 6 weeks. This will allow assessment of the effect of baseline predictors such as disability level, anxiety, depression, sleep self-efficacy level, and intervention adherence. The analysis will be conducted at the 5 % level of significance.

## 4 Discussion

Insomnia is a common problem in people with MS [4], which often remains unrecognized and therefore untreated [5]. CBT-I has been shown to be highly effective in addressing sleep disturbances in people with primary insomnia [19,20], as well as in populations of people with insomnia comorbid with a variety of chronic conditions [18]. However, evidence on the efficacy of CBT-I for pwMS remains limited. Our previous pilot studies demonstrated feasibility and large improvements in sleep outcomes in pwMS following CBT-I [28,29]. The proposed study represents the next needed step to determine the efficacy of CBT-I in this population. Specifically, we will conduct an adequately powered prospective randomized control trial (RCT) to assess the efficacy of CBT-I delivered via telehealth compared to an appropriate control group to improve sleep outcomes (Aim 1) and fatigue and health-related quality of life (Aim 2). An Exploratory Aim 3 will determine the participant characteristics that predict improvement in sleep outcomes. This will help guide health care providers and individuals with MS to select an appropriate sleep intervention at an individual level. Addressing insomnia symptoms using CBT-I could represent a low-cost, low-risk, non-pharmacological option for improving sleep quality, fatigue, and quality of life in individuals with MS and could result in reduced disability, improved quality of life, increased employment, and reduced costs associated with care and support for individuals with MS.

### 4.1 Anticipated challenges and limitations

Recruitment of individuals with a disability for clinical trials is often challenging. The proposed study will be conducted completely remotely across the United States which will increase the likelihood of successful recruitment. This will also enhance generalizability of the results. Another potential difficulty relates to the fact that individuals will enroll in this study for a period of 6 months which can pose a challenge to adherence to the principles learned from the intervention as well as retention in the study. In our recent pilot study of one-on-one in-person CBT-I in individuals with MS [28], retention was excellent; the CBT-I group had a retention rate of 83.3 % and adherence rate was or 93.3 %. Due to high comorbidity rates between depression and insomnia, excluding participants with severe depression can also be viewed as a limitation as this will likely restrict the variability in the range of scores for the Aim 3 analysis. Further, because individuals with untreated sleep disorders or elevated sleep apnea risk were excluded, findings may not generalize to people with MS who have co-morbid insomnia and sleep apnea. Although CBT-I can be effective in co-morbid insomnia and sleep apnea when obstructive sleep apnea is concurrently managed [55], this trial was not designed to assess or treat sleep apnea. Finally, exclusion of individuals with a history of overnight shift work within the last 5 years may be conservative, as long-term sleep disruption following shift work is not well established and it may therefore limit generalizability of the results.

## 5 Conclusion

While insomnia is a highly prevalent in individuals with MS, there is a critical need for an effective non-pharmacological intervention option to become more widely available. The current project will address this critical knowledge gap by addressing insomnia symptoms through CBT-I which will offer a low-cost, low-risk, non-pharmacological option for improving sleep quality and fatigue in individuals with MS.

## CRediT authorship contribution statement

**Catherine F. Siengsukon:** Writing – original draft, Supervision, Methodology, Conceptualization. **Jade Robichaud:** Writing – original draft, Investigation. **Eryen Nelson:** Writing – review & editing, Investigation. **Allison Glaser:** Writing – review & editing. **Garrett R. Baber:** Writing – review & editing, Investigation. **Matthew K.P. Gratton:** Writing – review & editing, Investigation. **Anna Zanotto:** Writing – original draft, Investigation. **Milind A. Phadnis:** Writing – review & editing, Methodology, Formal analysis. **Sharon Lynch:** Writing – original draft, Conceptualization.

## Funding statement

This work was supported by the 10.13039/100000890National MS Society [grant # RG-2307-41694].

## Declaration of competing interest

The authors declare the following financial interests/personal relationships which may be considered as potential competing interests:Dr. Siengsukon is the owner and CEO of Sleep Health Education, LLC.Dr. Lynch has participated in multicenter drug trials in Multiple Sclerosis with Novartis, Sanofi, Roche, Genentech, Bristol Myers Squibb, Contineum, Biogen, Atara, and Indapta. None of these drugs are associated with treatment of insomnia.

## Data Availability

No data was used for the research described in the article.
